# Identification of proteins expressed by *Babesia bigemina* kinetes

**DOI:** 10.1186/s13071-019-3531-7

**Published:** 2019-05-28

**Authors:** Gamila A. R. Bohaliga, Wendell C. Johnson, Naomi S. Taus, Hala E. Hussein, Reginaldo G. Bastos, Carlos E. Suarez, Glen A. Scoles, Massaro W. Ueti

**Affiliations:** 1 Program in Vector-borne Diseases, Department of Veterinary Microbiology and Pathology, Washington State University, Pullman, WA 99164 USA; 2 Animal Disease Research Unit, USDA-ARS, Pullman, WA 99164-6630 USA; 3 Department of Entomology, Faculty of Science, Cairo University, Giza, 12613 Egypt; 4 The Paul G. Allen School for Global Animal Health, Washington State University, Pullman, WA 99164-70403 USA

**Keywords:** *Babesia bigemina*, Kinete stage-specific protein, *in vitro* induced sexual stages, Kinete, *Rhipicephalus microplus*

## Abstract

**Background:**

*Babesia bigemin*a is an apicomplexan parasite transovarially transmitted *via Rhipicephalus* ticks that infect red blood cells and causes bovine babesiosis, a poorly controlled severe acute disease in cattle. New methods of control are urgently needed, including the development of transmission blocking vaccines (TBV). *Babesia bigemina* reproduces sexually in the gut of adult female *R. microplus* upon acquisition following a blood meal. Sexual reproduction results in zygotes that infect gut epithelial cells to transform into kinete stage parasites, which invade tick ovaries and infects the egg mass. The subsequent tick generation transmits *B. bigemina* upon feeding on bovine hosts. An important limitation for developing novel TBV is that the pattern of protein expression in *B. bigemina* tick stages, such as the kinete stage, remain essentially uncharacterized.

**Results:**

We determined the protein expression profile of three *B. bigemina* putative tick stage candidates BbiKSP (BBBOND_0206730), CCp2 and CCp3. We found that BbiKSP expression was restricted to *B. bigemina* kinetes. CCp2 and CCp3, previously shown to be expressed by induced sexual stages, were also expressed by kinetes. Importantly, none of these proteins were expressed by *B. bigemina* blood stages.

**Conclusions:**

*Babesia bigemina* kinetes express BbiKSP, CCp2 and CCp3 proteins, therefore, these proteins may play important roles during *B. bigemina* development within tick hemolymph and may serve as potential candidate targets for the development of TBV.

**Electronic supplementary material:**

The online version of this article (10.1186/s13071-019-3531-7) contains supplementary material, which is available to authorized users.

## Background

The apicomplexan parasite *Babesia bigemina* is an etiological agent of bovine babesiosis, a disease responsible for significant economic losses for the cattle industry. Bovine babesiosis is distributed globally and is transmitted transovarially by multiple Ixodidae tick species such as *Rhipicephalus microplus*, the major vector for *B. bigemina*. This parasite has a complex life-cycle that includes asexual reproduction while developing in bovine host erythrocytes and sexual reproduction in the midgut of the tick vector [1, 2]. The disease is poorly controlled and new vaccines are urgently needed. Ideally vaccines should be targeted at controlling both the development of debilitating or lethal acute disease (blood-stage vaccines), and transmission by ticks (transmission-blocking vaccines [TBV]). However, vaccine development is currently limited by our poor understanding of parasite biology, especially at the molecular level. This study is focused on defining proteins that are exclusively expressed in tick stages as candidates for future TBV development against *B. bigemina*. To produce effective strategies to block tick infection and parasite transmission, a comprehensive understanding of the parasite’s development in the tick vector is required.

The annotated genomes for several related arthropod-borne apicomplexan parasites such as *Plasmodium falciparum*, *P. vivax*, *B. bovis*, *B. bigemina*, *B. ovata* and *B.* sp. Xinjiang facilitated the identification of gene families conserved among apicomplexan parasites [3–8]. *In silico* and experimental data demonstrated that CCp2 and CCp3 are well conserved among arthropod-borne apicomplexan parasites including *Plasmodium*, *Theileria* and *Babesia* and are consistently expressed in *in vitro* induced sexual stages [9, 10]. Transcription of tick stage-specific genes, and associated protein expression, of *B. bigemina* has recently been documented during the parasite’s development in the vector [11]. Bohaliga et al. [11] identified BBBOND_0204030 as a gene uniquely transcribed by *B. bigemina* tick stages and by *in vitro* induced *B. bigemina* sexual stages. The *B. bigemina* HAP2 protein, that is associated with the development of gametes in apicomplexan parasites [12], was found to be expressed by parasite stages developing in the midgut of infected ticks at day 3 post-repletion of female *R*. *microplus* ticks. In addition, studies identified and demonstrated protein expression of *B. bigemina* CCp1-3, a gene family also related to the development of sexual stages in apicomplexans [10], by *B. bigemina in vitro* induced sexual stages but not blood stages [10, 11]. Finally, Johnson et al. [13] identified kinete stage-specific genes that are highly transcribed by *B. bovis* and *B. bigemina* kinetes as compared to blood stages. A *B. bovis* protein identified as BBOV_I002220 was specifically expressed by kinetes and is homologous to the protein encoded by *B. bigemina* BBBOND_0206730 gene (hereby renamed as BbiKSP, *B. bigemina* kinete stage-specific protein) [13]. In this study, we report for the first time the unique expression of BbiKSP by *B. bigemina* kinetes. In addition, we demonstrate that CCp2 and CCp3 proteins, previously shown to be expressed by *in vitro* induced sexual stages, are also expressed by kinetes isolated from the hemolymph of *B. bigemina*-infected adult female *R. microplus* ticks.

## Methods

### *In silico* analysis

*Babesia bigemina* tick specific genes were selected based on amino acid identity to *P. falciparum*, *P. vivax* and *B. bovis* [3–5]. The available annotated *B. bigemina* genome was used to determine features of selected genes [6]. The amino acid sequences of *P. vivax* Sal-1 strain CCp2 (GenBank: XP_001615829), *P. falciparum* 3D7 strain CCp3 (GenBank: XP_001348240), and *B. bovis* T2Bo strain BBOV_I002220 (GenBank: XP_001608872.1) were used to blast against the *B. bigemina* genome [6] using BLASTp software (https: //blast.ncbi.nlm.nih.gov/Blast). Clustal omega analysis (http://www.ebi.ac.uk/Tools/msa/clustalo/) was used to determine the percent amino acid identity of proteins. The Simple Modular Architecture Research Tool (SMART) (http://smart.embl-heidelberg.de/) was used to identify predicted conserved domains. Presence of signal peptide, transmembrane domains and exterior protein presentation were evaluated using SignalP 4.1 server (http://www.cbs.dtu.dk/services/SignalP/), TMHMM (http://www.cbs.dtu.dk/services/TMHMM/) and PSIPRED (http://bioinf.cs.ucl.ac.uk/psipred/), respectively.

### Isolation of *B. bigemina* kinetes from *R. microplus* adult female ticks

To isolate *B. bigemina* kinetes from infected *R. microplus* adult female ticks, a naive splenectomized Holstein calf, four months of age and tested to be bovine babesiosis-free by nested PCR and competitive enzyme-linked immunosorbent assay [14, 15] was used for feeding an estimated 40,000 *R. microplus* larvae applied under a cloth back patch. After 14 days, when approximately 1% of the ticks had molted to adults, 10^7^ *B. bigemina* Mexico strain infected erythrocytes were inoculated intravenously to synchronize tick acquisition feeding with an ascending *B. bigemina* parasitemia. Replete female ticks were collected at days 7 to 9 post-infection. Ticks were rinsed in tap water and placed into individual 24-well tissue culture plates and incubated at 26 °C in airtight containers with saturated KNO_3_ solution at 92% relative humidity for 6 days to allow accumulation of *B. bigemina* kinetes in the tick hemolymph. Ticks infected with *B. bigemina* were identified by removing the distal leg segment of engorged female ticks and exuding hemolymph onto slides. Slides were stained with Giemsa and examined by light microscopy for the presence of the parasite. *Babesia bigemina* kinetes were collected from infected female *R. microplus* ticks, as previous described [16]. In brief, ticks were placed on double-sided tape with the ventral side up. The cuticle was perforated using a 26 gauge needle and approximately 200 μl of Hank’s Balanced Salt Solution (HBSS) (Thermo Fisher Scientific, Waltham, MA, USA) injected into the membrane surrounding the base of the fourth leg coxal using a 33 gauge, 12.7 by 0.21 mm needle. Kinetes were collected by extraction of hemolymph-containing fluid with negative pressurized capillary tubing [16]. Hemolymph samples were pooled and the kinetes concentrated by centrifugation at 4000×*g* for 2 min. Kinetes were used for RNA extraction and immunofluorescence assay (IFA) antigen.

### RNA isolation and cDNA synthesis

Incubated replete female ticks were dissected daily from day 0 through day 6 post-incubation. Tick midgut and kinetes were collected in Trizol (Thermo Fisher Scientific) and stored at −80 °C. Total RNA isolation and DNase-Free treatment were performed following the manufacturer’s protocol. One hundred ng of total RNA was utilized for cDNA synthesis using a Superscript III™ cDNA Synthesis Kit (Thermo Fisher Scientific) following the manufacturer’s protocol. Synthesized cDNA was used in reverse transcription PCR (RT-PCR) to detect *B. bigemina* transcripts. Primer sets were designed based on *B. bigemina* genome sequences for BbiKSP (Forward 5’-GTG CAA AGC TGG TTG AAG AC-3’ and Reverse 5’-GCA TGG ATA TCG TAC TGG TGT AG-3’) and actin (BBBOND_0107357) (Forward 5’-ATC GCC GTT TAC ACT TCA CG-3’ and Reverse 5’-GCC CCT TCC TCC TCG TAA TC-3’). *Rhipicephalus microplus* α-tubulin primers (Forward 5’-CGT GCC GTA TTT GTT GAT C-3’ and Forward 5’-AGA TTA GCT GCT CCG GGT G-3’) were used as previously described [17]. The predicted amplicon sizes are 493 bp, 131 bp and 91 bp, respectively. RT-PCR reactions were conducted in 20 μl containing 2 μl of synthesized cDNA, 1 µl of 10 µM of each primer, 6 µl of nuclease-free water and 10 µl of RedTaq (Sigma-Aldrich, Saint Louis, MO, USA). The amplification conditions consisted of 3 min denaturation at 95 °C, 35 repeated cycles of 30 s denaturation at 95 °C, 30 s annealing at 55 °C and 30 s extension at 72 °C, with a final 7 min extension at 72 °C. Amplicons were resolved using 1% agarose gel electrophoresis. All PCR products were sequenced (Eurofins Genomics, Louisville, KY, USA).

### *In vitro* induction of *B. bigemina* sexual stages

*Babesia bigemina* sexual stages were produced as previously described [11]. In brief, *B. bigemina* infected erythrocytes were grown in HL-1 medium (Lonza, Walkersville, MD) supplemented with 40% normal bovine serum, 10 mM 3-[N-tris (hydroxymethyl) methylanino]-2-hydroxypropanesulfonic acid (Sigma-Aldrich, St. Louis, MO, USA), and antibiotic/antimycotic (Sigma-Aldrich, St. Louis, MO, USA), pH 7.2, with a 5% packed cell volume of bovine red blood cells. To increase parasitemia, cultures were incubated at 37 °C in 5% CO_2_ and expanded without addition of erythrocytes [18]. When the parasitemia exceeded 10%, *B. bigemina* sexual stages were induced with 20 mM tris(2-carboxyethyl)phosphine (Thermo Fisher scientific) and incubated for 1 h at 37 °C with 5% CO_2_ as previously described [11]. Cultures were then washed once with fresh medium to remove excess reducing agent by centrifugation for 3 min at 2655×*g*. Parasites were cultured in fresh medium and incubated for up to 24 h at 37 °C with 5% CO_2_. To analyze the formation of sexual stages, smears were made from all cultures at 6, 15 and 24 h post-induction and stained with Hema 3 stain (Thermo Fisher Scientific). Cultures were collected at 0, 6, 15 and 24 h post-induction for RNA extraction and IFA antigens. Total RNA isolation, DNase-free treatment and cDNA synthesis from non-induced and *in vitro* induced cultures at 6, 15 and 24 h were performed as described above. cDNA at different time points were used in RT-PCR to detect the transcription of the genes of interest.

### Production of bovine antiserum

To characterize *B. bigemina* protein expression by kinetes isolated from the hemolymph of *R. microplus* adult female ticks, rabbit antisera were raised against CCp2 and CCp3 as previously described [10] and bovine antiserum was raised for BbiKSP. To generate bovine antisera against BbiKSP, synthetic peptides ranging from 13 to 20 amino acids were selected based on a proprietary algorithm identifying surface exposed B cell epitopes (BioSynthesis, Inc. Lewisville, TX, USA) using the sequence of *B. bigemina* BbiKSP. Three peptides were chosen for immunization: peptide1-KNQKTAIQDQRKDVDAKSKT (aa 117–136); peptide2-CQRHMPTERRDTN (aa 290–302) and peptide3-ALKPEETEDSGKES (aa 580–593). A single spleen intact naive calf was subcutaneously immunized with individual preparations of each peptide in different draining lymph nodes. Each inoculation contained 50 µg of a peptide conjugated to KLH plus 750 µg of Quil-A Saponin adjuvant (Invivogen, San Diego, CA, USA). The calf received four booster inoculations at 3 week intervals. Before the first immunization and three weeks after the final immunization, serum was collected. For control sera, a second calf was vaccinated with KLH in Quil A Saponin using the same protocol. Animals were euthanized based on the euthanasia protocol approved by the Institutional Animal Care and Use Committee (IACUC).

### Immunofluorescence assays

Fixed IFA were performed as previously described [10, 11, 13]. In brief, non-induced and *in vitro* induced *B. bigemina* cultures were collected at 0, 6, 15, and 24 h and pelleted at 3000×*g* for 15 min at 4 °C. Cells were washed two times with 1× PBS at 3000×*g* for 15 min at 4 °C and pellets suspended in PBS containing 3% bovine serum albumin (BSA). These cells were used to make smears on glass microscope slides. *Babesia bigemina* kinetes were collected as described above and washed once with 1× PBS at 4000× *g* for 2 min at 4 °C. The pellets were suspended in 1× PBS containing 3% BSA and deposited into individual wells of Teflon coated slides and air dried. Slides were fixed in cold acetone for 1 min and blocked with 1× PBS containing 10% goat normal serum (PBS-NGS) and incubated for 30 min at 37 °C in a humidity chamber. Slides were rinsed with ddH_2_O and air dried. Rabbit anti CCp2, anti-CCp3 [10] and bovine anti-BbiKSP or anti-KLH primary antisera were diluted 1:20 in PBS-NGS and applied to individual wells. Slides were placed in a humidity chamber for 30 min at 37 °C. Then slides were rinsed once with ddH_2_O and washed three times for 10 min with cold 1× PBS. The slides were incubated at 37 °C for 30 min with either goat anti-rabbit IgG-Alexa Fluor 647 (Thermo Fisher Scientific) conjugated secondary antibody diluted 1:1000 in PBS-NGS for reactions to CCp2-3 antigen or goat anti-bovine IgG-FITC (SeraCare, Gaithersburg, MD, USA) conjugated secondary antibody diluted 1:100 in PBS-NGS for reactions to BbiKSP or KLH. All samples were washed twice in 1× PBS, once with ddH_2_O and air dried. Finally, one drop of ProLong Gold anti-fade reagent with DAPI (Invitrogen, Eugene, OR, USA) was added to each sample under a coverslip. Identically produced negative controls were performed using pre-immune rabbit, pre-immune bovine or bovine anti-KLH serum as the primary antibody. All samples were visualized under a Leica microscope (Buffalo Grove, IL, USA) using LAS-X software.

## Results

### *In silico* analysis

In this study we focused on three proteins termed CCp2 and CCp3, previously identified as vector-specific proteins in other apicomplexa [10] and BbiKSP (Table 1). Bioinformatic analysis was performed to determine predicted features of the selected *B. bigemina* proteins such as molecular weight, signal peptide, and transmembrane domains (Table 2). Using this approach, we demonstrated that BbiKSP has a signal peptide at cleavage site between position 17 and 18. BbiKSP did not have any other identifiable conserved or transmembrane domains. Blasting BbiKSP against *Babesia*, *Theileria*, and *Plasmodium* species suggests BbiKSP is exclusively conserved within transovarially transmitted *sensu stricto Babesia* species as homologs of this gene were not found in intrastadial or transstadial transmitted parasites, including *Plasmodium* or *Theileria* (Table 3 and Additional file 1: Figure S1).

**Table 1:** Accession numbers of *B. bigemina* selected proteins and their homologous proteins in other arthropod-borne pathogens

Protein name	*B. big*	*B. bov*	*B. ova*	*B.* sp. Xin	*B. m*	*T. a*	*T. e*	*T. p*	*Pl*
BbiKSP	XP_012767701	XP_001608872	GBE61312	ORM40592	–	–	–	–	–
CCp2	XP_012767423	XP_001609893	GBE61548	ORM40789	XP_012649113	XP_952882	XP_004830733	XP_763844	XP_001615829
CCp3	XP_012769523	XP_001612018	GBE58642	ORM39994	XP_021337760	XP_953429	XP_004831308	XP_764433	XP_001348240

*Abbreviations*: *B. big*, *Babesia bigemina*; *B. bov*, *Babesia bovis*; *B. ova*, *Babesia ovata*; *B.* sp. Xin, *Babesia* sp. Xinjiang; *B.m*, *Babesia microti*; *T.a*, *Theileria annulata*; *T.e*, *Theileria equi*; *T.p*, *Theileria parva*; *Pl*, *Plasmodium* species

**Table 2:** Predicted features of the selected *B. bigemina* proteins

Protein	Chromosome	Exons	Signal peptide	Conserved domain	Nucleotide/Amino acid	MW (KDa)
BbiKSP	2	1	Yes	None	1839/612	67.48
CCp2	2	8	No	LCCL	4893/1630	178.74
CCp3	3	16	Yes	LCCL	3798/1265	137.20

**Table 3:** *B. bigemina* selected proteins and protein identities to other arthropod-borne pathogens

*B. bigemina* protein name	Accession number	% amino acid identity							
		*B. bov*	*B. ova*	*B. sp. Xin*	*Bm*	*Ta*	*Te*	*Tp*	*Pl*
BbiKSP	XP_012767701	22.94	65.47	28.50	–	–	–	–	–
CCp2	XP_012767423	62.78	91.30	70.65	26.71	42.51	45.77	42.52	32.03
CCp3	XP_012769523	72.85	92.60	80.78	31.29	52.70	52.72	53.00	37.03

*Abbreviations*: *B. bov*, *Babesia bovis*; *B. ova*, *Babeisa ovata*; *B.* sp. Xin, *Babesia* sp. Xinjiang; *Bm*, *Babesia microti*; *Ta*, *Theileria annulata*; *Te*, *Theileria equi*; *T.p*, *Theileria parva*; *Pl*, *Plasmodium* species

### Pattern of transcription of BbiKSP between *Babesia bigemina* blood and tick midgut stages

**Fig. 1:**
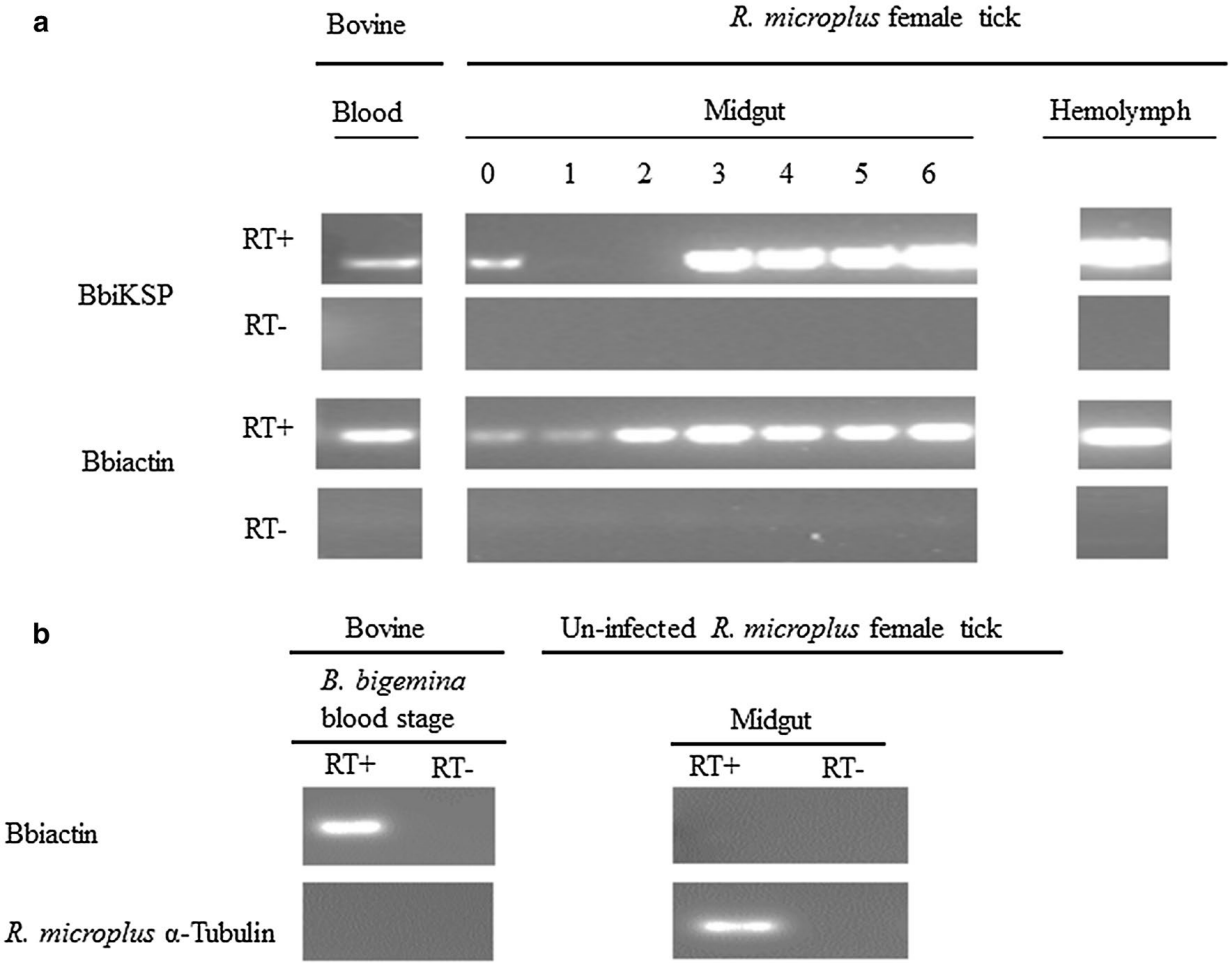
Detection of BbiKSP transcripts during *B. bigemina* development in the mammalian and tick hosts. (**a**) Blood, midguts from day 0 to 6 post-incubation of engorged female ticks and infected hemolymph. To demonstrate that Bbiactin was specific for *B. bigemina* (**b**) *B. bigemina* actin was detected in infected blood but not in midgut from uninfected female tick. *R. microplus* α-Tubulin was used to demonstrate tick RNA quality. RT− and RT+ indicate the absence or presence of reverse transcriptase

**Fig. 2:**
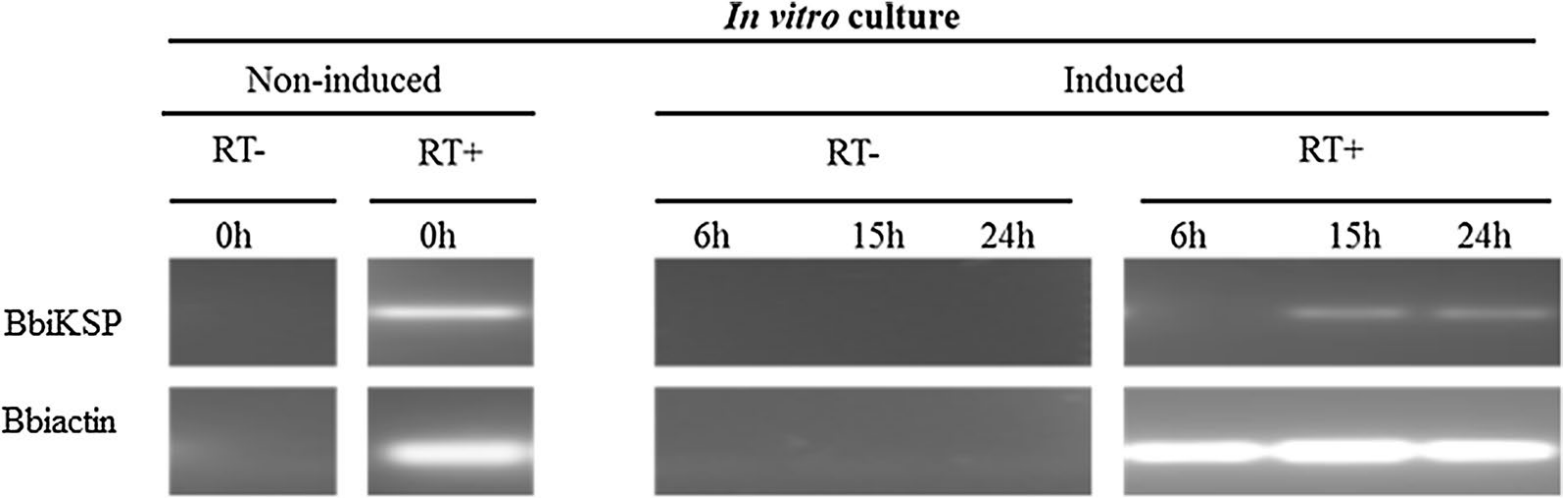
Detection of BbiKSP transcripts in non-induced blood and *in vitro* induced cultures at multiples time points. Bbiactin was used as a reference gene. RT− and RT+ indicate the absence or presence of reverse transcriptase

RT-PCR analysis demonstrated that BbiKSP is transcribed in both *B. bigemina* blood and tick stages. BbiKSP transcripts were detected at various time-points in *B. bigemina* infected tick gut, as well as in kinetes from hemolymph (Fig. 1a). RT**-**PCR analysis for BbiKSP performed on tick midgut RNA samples displayed an on/off pattern, with the day 0 signal providing results similar to blood stages. Days 1–2 midgut samples retained a *B. bigemina* actin signal but did not show a BbiKSP signal. Beginning at day 3, all further midgut sampling dates were positive for BbiKSP. The specificity of Bbiactin primers was confirmed using cDNA made from *B. bigemina*-infected blood and uninfected midgut female tick RNA (Fig. 1b). The quality and integrity of tick cDNA was confirmed by RT-PCR amplification of *R. microplus* α-Tubulin (Fig. 1b). RT(−) controls for all tissues were performed and did not yield any detectable product indicating the lack of genomic DNA in the samples. In addition, BbiKSP mRNA was transcribed by non-induced and *in vitro* induced cultures of *B. bigemina* at 15 and 24 h of post-induction but not 6 h post-induction (Fig. 2). These *in vitro* induction results were fully consistent with the *ex vivo* observation described above. All RT-PCR products obtained in these experiments were sequenced, and the amplicon identities confirmed.

### Analysis of protein expression by *B. bigemina* kinetes

**Fig. 3:**
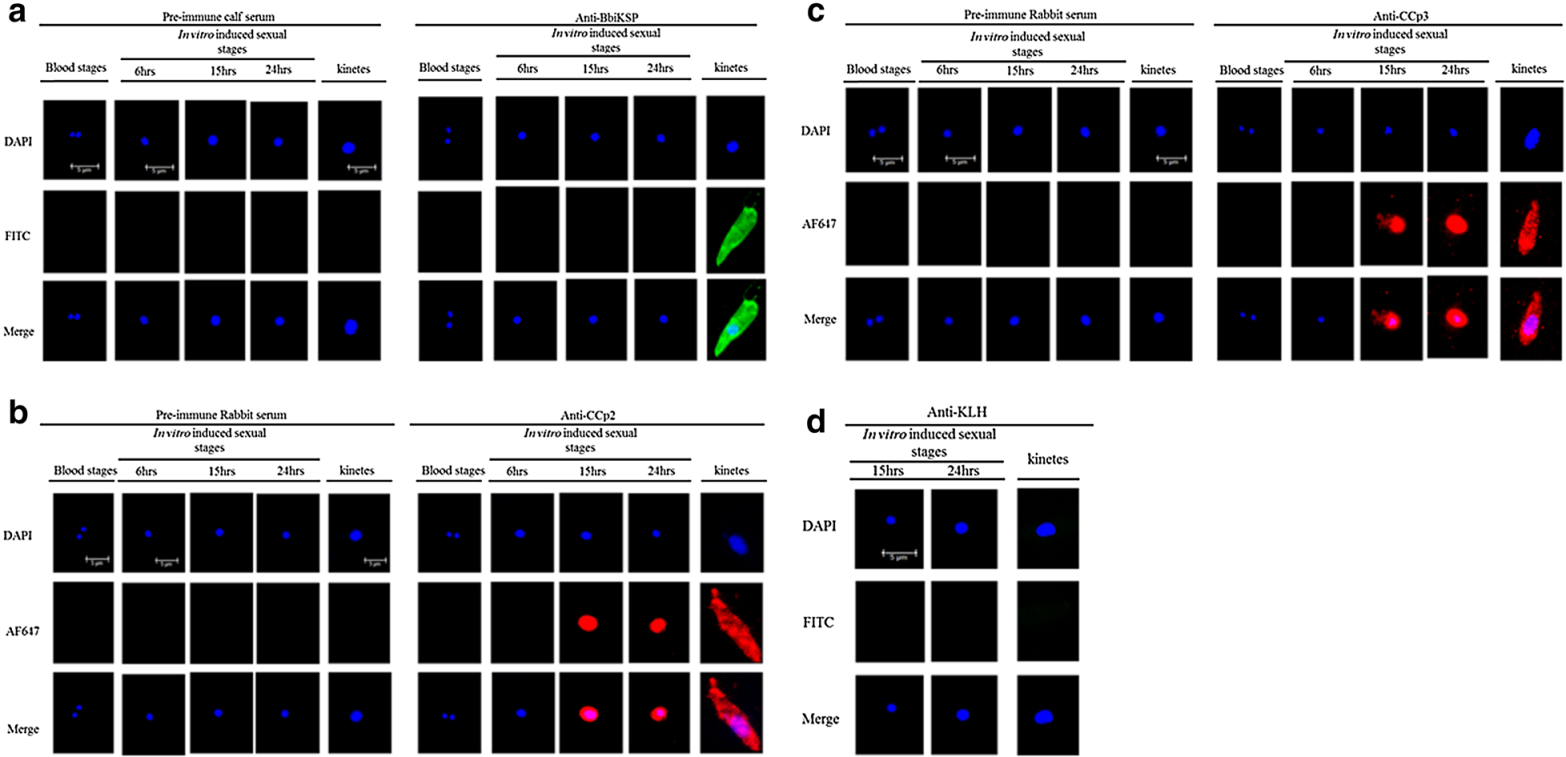
Immunofluorescence assays demonstrating expression of BbiKSP (**a**), CCp 2 (**b**), CCp3 (**c**) by *B. bigemina* kinetes but not blood stages and bovine anti-KLH as control (**d**). *Scale-bars*: 3 and 5 µm

Protein expression of BbiKSP was only detected by IFA in *B. bigemina* kinetes and not in blood stages or *in vitro* induced sexual stages (Fig. 3a). Control reactions using antibodies against the peptide carrier KLH were negative for both *in vitro* induced sexual stages and kinetes, suggesting that the antibodies react specifically with the target proteins (Fig. 3d). For *in vitro* induced sexual stages, anti-CCp2 and CCp3 antibodies were used as positive controls for the IFA (Fig. 3b, c). However, CCp2 and CCp3 expression was not limited to sexual stages as a strong IFA reaction for both proteins to kinetes was observed (Fig. 3b, c). Blood stages did not express either CCp protein (Fig. 3b, c). The reactions to all proteins did not appear to be confined to a particular region or organelle within the parasite, instead the reactions were uniform across the entire parasite. Table 4 summarizes the expression profiles of BbiKSP, CCp2 and CCp3.

**Table 4:** Summary of expression profiles of BbiKSP, CCp2 and CCp3

Expression profile	Blood stages	Induced sexual stages			Kinetes
		6 h	15 h	24 h	
BbiKSP					
mRNA	+	−	+	+	+
Protein	−	−	−	−	+
CCp2					
mRNA	+	+	+	+	+
Protein	−	−	+	+	+
CCp3					
mRNA	+	+	+	+	+
Protein	−	−	+	+	+

## Discussion

*Babesia bigemina* has a complex life-cycle that includes asexual reproduction in the bovine host and sexual reproduction in tick vectors [1, 2]. When adult female ticks feed on an animal infected with *B. bigemina*, the parasite sexually reproduces within the biological vector, undergoes several morphological changes, and interacts with multiple cell types and tissues including tick midgut, ovaries and salivary glands before transmission [1, 2]. Proteins that are expressed by tick stages of *B. bigemina* remain uncharacterized.

In this study, we demonstrated that BbiKSP mRNA was transcribed in the stages of the parasite present in infected bovine blood and infected tick midgut and hemolymph. However, BbiKSP polypeptides were not detected in either blood stages or *in vitro* induced sexual stages, including *B. bigemina* gametocytes, gametes and zygotes. This is in agreement with a previous finding demonstrating expression of its *B. bovis* homologous protein BboKSP, BBOV_I002220, exclusively in *B. bovis* kinetes despite the presence of transcripts, albeit at low levels, in infected blood [13]. Our comparative *in silico* data demonstrated that BbiKSP was conserved among *Babesia* parasites including *B. bovis*, *B. ovata* and *B.* sp. Xinjiang (Additional file 1: Figure S1), with the greatest identity, 65.47%, shared between *B. bigemina* and *B. ovata*. Previously, BboKSP, which contains a C terminal GPI anchor, was characterized with cell surface staining techniques to suggest it was on the exterior surface of the kinete [13]. No homologs of BbiKSP were found in the genomes of the closely related *Plasmodium* and *Theileria* parasites, nor in *Babesia* (*sensu lato*) parasites such as *B. microti*. While these three parasites species lacking a KSP gene undergo sexual reproduction in their arthropod vectors, none are transovarially transmitted. In contrast, the data suggest that all transovarially transmitted *Babesia* (*sensu stricto*) species, such as *B. bovis*, contain genes that are homologous to BbiKSP [7, 8, 13]. It is possible that BbiKSP encodes for a protein involved in parasite invasion into the ovaries and, thus, is required for transovarial transmission.

*In silico* predictions of BbiKSP cellular localization by TMHMM and PSIPRED indicated that the protein is extracellular. This feature is compatible with the premise that the protein interacts with host moieties to invade the invertebrate host cells. The nature of this interaction is unknown, however, a possible mechanism that could facilitate transovarial transmission of the parasite might involve BbiKSP and vitellogenin. Kinetes develop from parasite zygotes that have invaded basophilic cells in the tick midgut. Basophilic cells are major producers of vitellogenin, a lipoglycoprotein that is used to form the yolks of developing eggs [19]. RNAi experiments interfering with vitellogenin receptors (VgR) have abolished transovarial transmission of *B. bovis* in *R. microplus* ticks [20]. The fixed IFA results for BbiKSP are consistent with an exterior membrane-associated location, however, confirmation of the extracellular display of BbiKSP awaits further testing using live, intact *B. bigemina* kinetes.

Additionally, we demonstrated that CCp2 and CCp3 were expressed by *B. bigemina* kinetes. Members of the CCp family have been previously identified and characterized by related apicomplexan parasites including *Plasmodium, Theileria* and *Babesia* [9, 10, 21]. The presence of CCp orthologous proteins in related apicomplexan parasites suggests potentially conserved functions for this family. Our data suggest that *B. bigemina* CCp2 and CCp3 proteins may also play a role in *B. bigemina* transovarial transmission.

A surprising finding in this study is the presence of RNA transcript without translated polypeptide. This has been described before for *B. bigemina* CCp 1-3 [10] and, as mentioned previously, for BboKSP [13]. Interestingly, a similar lack of correlation among RNA and protein levels was previously reported for the members of the RAP-1 gene family in *B. bigemina* and other rhoptry proteins expressed by *Plasmodium* parasites. It was postulated that these findings may reflect a gene expression regulatory mechanism used by the parasite to adapt to variations in life- cycle, such as the different vertebrate and invertebrate vector environments [22]. Gene expression is controlled at many different stages and by many different mechanisms, thus, establishing correlations between mRNA and protein levels are not always possible. There are cases where transcript copy number strongly correlates with protein levels; however, regulation of gene expression may be controlled upstream of transcription and/or translation, resulting in poor correlation between mRNA and protein levels. Post-transcriptional modifications of mRNA and the activity of microRNAs or other regulatory molecules may be the reasons for low or undetectable protein expression despite having corresponding high mRNA levels in a cell [23]. Some of these regulatory mechanisms may be operating as regulons for the synthesis of proteins in *Babesia* parasites, but unfortunately, these regulatory mechanisms remain unexplored in most apicomplexan parasites and at this point we can only speculate on the possible explanations.

Regardless of the translational control mechanism involved, it is interesting to note that mRNA for BbiKSP was not detected in midguts from days 1–2 of replete, incubated females before returning to detectable levels on day 3. The pattern of BbiKSP transcription indicates significant changes in transcriptional regulation for this gene during the early development of the parasite in the vector. This is consistent with our induced sexual stage results that showed an initial loss of BbiKSP transcription signal at 6 h that subsequently returned at 15 h. We postulated that sexual stages developing in tick midgut undergo a similar change in transcription patterns. This is supported by our previous work that demonstrated a five log increase in transcript for BbiKSP in kinetes as compared to blood stages [13]. Apicomplexa use a suite of transcription control factors known as AP2 that differentially regulate gene expression. Specific AP2, such as the described AP2-G in *Plasmodium* gametes, direct gene expression in a life-cycle stage dependent fashion [24]. A recent review showed that *Babesia* also have AP2 and that they possess a homolog for AP2-G [25]. The return of BbiKSP transcripts by day 3 in replete females with concomitant polypeptide expression by circulating kinetes suggests a turnover in which AP2 are directing gene transcription. This may also include genes other than BbiKSP that ensure the complete translation of the mRNA and appropriate cellular display. The stage-specific expression of BbiKSP appears to be similar to 6-Cys-C that is restricted to the kinete stage of *B. bovis* [26]. In contrast, polypeptide expression of genes important during *B. bigemina* blood stages, such as Rhoptry Associated Protein-1a (RAP-1a), can be detected by IFA throughout the entire life-cycle of the parasite. However, RAP-1a does not appear to localize to a rhoptry organelle in kinetes but, rather, is distributed in a speckled pattern within the parasite [27]. This resembles the inappropriate cellular partitioning that occurs with ROP2 in the absence of adaptor complex 1 in *Toxoplasma gondii* [23], reinforcing the suggestion that secondary signals are required by apicomplexans for complete translation and display of surface proteins. It is also possible that expression of the *Babesia* KSPs described hereby are also subjected to such finely regulated mechanisms, resulting in the expression of KSP proteins only when and where they are required for a function that is related to the invasion of ovary tissues of the tick, in order to allow occurrence of the transovarial transmission mechanisms typical of *Babesia* (*sensu stricto*) parasites.

## Conclusions

*Babesia bigemina* is the one of most important parasites responsible for bovine babesiosis that results in a high negative economic impact on the cattle industry. To improve bovine babesiosis control, understanding the events of parasite development within the vector and characterizing parasite proteins expressed by parasite tick stages is crucial. In this study, we document BbiKSP expression as unique to the kinete stage. In addition, CCp2 and CCp3 proteins were found to be expressed by kinetes. The tick stage-specific expression of these parasite proteins, and their possible involvement in the mechanism of transovarial transmission, strongly suggest their future use as potential candidates for developing *Babesia* transmission blocking vaccines. Further investigations are needed to determine the role and function of these proteins during the parasite development within adult female ticks.

## Additional file

**Additional file 1: Figure S1.** Amino acids alignment of BbiKSP among *Babesia* species with their accession numbers: *B. bigemina*, XP_012767701.1; *B. bovis*, XP_001608872.1; *B. ovata*, GBE61312.1; *B. sp. Xinjiang*, ORM40592.1.

## Data Availability

The data supporting the conclusions of this article are included within the article and its additional files.

## Abbreviation

PBS: phosphate-buffered saline
