# Supplementary material for: Identification of proteins expressed by Babesia bigemina kinetes
Source: Parasit Vectors. 2019 May 28;12:271. doi: 10.1186/s13071-019-3531-7 (PMC6537212; doi:10.1186/s13071-019-3531-7)
Supplement: Supplementary file 1 — Additional file 1: Figure S1. Amino acids alignment of BbiKSP among Babesia species with their accession numbers: B. bigemina, XP_012767701.1; B. bovis, XP_001608872.1; B. ovata, GBE61312.1; B. sp. Xinjiang, ORM40592.1. [file 13071_2019_3531_MOESM1_ESM.docx]

**Additional file 1: Figure S1.** Amino acids alignment of BbiKSP among *Babesia* species with their accession numbers: *B. bigemina*, XP_012767701.1; *B. bovis*, XP_001608872.1; *B. ovata*, GBE61312.1; *B. sp.* *Xinjiang*, ORM40592.1.
